# Genotypes and virulence in serotype K2 *Klebsiella pneumoniae* from liver abscess and non-infectious carriers in Hong Kong, Singapore and Taiwan

**DOI:** 10.1186/1757-4749-6-21

**Published:** 2014-06-12

**Authors:** Jung-Chung Lin, Tse Hsien Koh, Nelson Lee, Chang-Phone Fung, Feng-Yee Chang, Yu-Kuo Tsai, Margaret Ip, L Kristopher Siu

**Affiliations:** 1 Division of Infectious Diseases and Tropical Medicine, Department of Internal Medicine, Tri-Service General Hospital, National Defense Medical Center, Taipei, Taiwan; 2 Department of Pathology, Singapore General Hospital, Singapore, Singapore; 3 Department of Medicine and Therapeutics, Faculty of Medicine, Chinese University of Hong Kong, Hong Kong, SAR, People’s Republic of China; 4 Department of Medicine, Section of Infectious Diseases, Taipei Veterans General Hospital and National Yang-Ming University, Taipei, Taiwan; 5 National Institute of Infectious Diseases and Vaccinology, National Health Research Institutes, 35 Keyan Road, Zhunan, Miaoli County 350, Taiwan; 6 Department of Microbiology, Faculty of Medicine, Chinese University of Hong Kong, Hong Kong, SAR, People’s Republic of China; 7 Graduate Institute of Basic Medical Science, China Medical University, Taichung, Taiwan

**Keywords:** Liver abscess, *Klebsiella pneumoniae*, MLST

## Abstract

In *Klebsiella pneumoniae* liver abscess (KP-LA), *K. pneumonia*e K2 is the most frequently isolated serotype after K1, but this serotype has been much less studied. In the present study, the molecular types sequences type (MLST) of serotype K2 isolates from three different regions in Asia were identified and the virulence of these isolates was investigated. Eight different MLSTs were found among 26 isolates (ST 65, 66, 86, 373, 374, 375, 380, and 434). There were two major MLST groups, ST-65-like (42%) and ST86-like (46%). No isolates contained *allS* while all isolates contained *rmpA*. The prevalence of aerobactin gene and *kfu* were 25/26 (96%) and 3/26 (11.5%) respectively. Although liver abscess isolates were generally more resistant (11/15 isolates) to serum killing, there was no specific distribution of serum killing resistant or susceptible ST types between stool carriage and liver abscess isolates. Neutrophil phagocytosis showed that the liver abscess and carriage isolates varied in their susceptibility to phagocytosis. Strains with resistance to both neutrophil phagocytosis and serum killing were generally hypervirulent with lethality at LD_50_ < 10^3^ colony forming units by intraperitoneal injection. In conclusion, Anti-phagocytosis and resistance to serum killing are two parameters that most predict hyperviurlence in serotype K2 isolates. Unlike serotype K1 KP-LA that mainly belong to ST-23, ST-65-like and −86-like are the two major MLST types among serotype K2 isolates from Singapore, Hong Kong and Taiwan.

## Introduction

*Klebsiella pneumoniae* liver abscess (KP-LA) has becoming a global emerging disease [1,2]. The etiology of this disease has been investigated by different study groups [1,3-5]. Several bacterial virulence factors have been investigated and virulence has often been found to be capsule related [2,6,7]. *K. pneumoniae* serotype K1 causing liver abscesses LA has been observed worldwide and is the most prevalent type among all 77 serotypes [1,6,8]. Previous molecular and virulence analysis in *K. pneumoniae* serotype K1 has shown that ST23 was predominant in serotype K1 *K. pneumoniae* isolates causing liver abscess and carried in stools of uninfected subjects in Hong Kong, Singapore and Taiwan [9]. In addition, serotype K1 isolates with ST23 could have different mice lethal dose (LD_50_) indicating normally expressed K1 capsule is not the sole factor for hypervirulence but its phagocytic resistance and carriage of the aerobactin gene were two independent determinants contributing to mouse lethality [2,9]. Although *kfu* (gene encoding an iron uptake system) and *allS* (a gene associated with allantoin metabolism) have been documented as virulence factors contributing to virulence, all invasive serotype K1 isolates contained these two determinants in a previous study [10-12].

Although non-K1 serotypes have been observed in KP-LA, they were less frequently encountered. Serotype K2 KP is the second most commonly isolated serotype in KP-LA in Taiwan and has also been reported in Asia and US [1,13-15]. Relatively few studies have specifically focused on this serotype. Little is known about the STs and the prevalence of virulence factors in serotype K2 causing KP-LA in different countries.

In this study, we determined the molecular types (MLST) of *K. pneumoniae* serotype K2 isolates from liver abscesses and from carriers without a history of KP-LA and assessed the virulence of isolates with different MLST types from Hong Kong, Singapore, and Taiwan.

## Material and methods

### Bacterial strains

*K. pneumoniae* strains that were isolated from liver abscess and stool from healthy subjects, hospitalized patients without history of liver abscess or patients admitted with noninfectious diseases were collected at Prince of Wales hospital in Hong Kong, Singapore General Hospital, National University hospital in Singapore and Tri-Service General Hospital in Taiwan from 2002 to 2009. One isolate was collected from each patient will liver abscess, healthy subjects, hospitalized patients without history of liver abscess or patients admitted with noninfectious diseases. The diagnosis of liver abscess was confirmed by abdominal ultrasonography and/or computerized tomography. Identification of the isolates was according to standard clinical microbiologic methods.

### Serotyping, *kfu, alls, rmpA* and aerobactin gene detection by PCR

Isolates were serotyped by PCR as previously described [16]. PCRs to determine the presence of the specific genes for serotype K1, K2 and K5 [17], *rmpA*, *alls, kfu* and the aerobactin gene [5] were performed using primers as listed in Table 1. A bacterial colony from an overnight-culture was added to 300 μl water and boiled for 15 min to release DNA template. The reaction mixture was kept at 95°C for 5 min, followed by 40 temperature cycles of 95°C for1 min, 50°C for 1 min, and 72°C for 2 min, and 72°C for 7 min. The expected PCR products were 641 bp for *wzy*_KPK2_, 535 bp for *rmpA*, 520 bp for *kfu*, 508 bp for *allS* and either 556 or 531 bp for aerobactin in length (Table 1).

**Table 1:** **Specific primers used for amplification of the target genes of *K. pneumoniae *** **in this study**

**Serotype (target gene)**	**Primer**	**Size of PCR product (bp)**	**Reference**
K1 (*wzy*_KPK1_)	5′-GGTGCTCTTTACATCATTGC-3′	1283	[17]
	5′-GCAATGGCCATTTGCGTTAG-3′		
K2 (*wzy*_KPK2_)	5′-GACCCGATATTCATACTTGACAGAG-3′	641	[17]
	5′-CCTGAAGTAAAATCGTAAATAGATGGC-3′		
K5 (*wzx*_KPK5_)	5′-TGGTAGTGATGCTCGCGA-3′	280	[17]
	5′-CCTGAACCCACCCCAATC-3′		
*RmpA*	5′-ACTGGGCTACCTCTGCTTCA-3′	536	[5]
	5′-CTTGCATGAGCCATCTTTCA-3′		
Aerobactin	5′-GCATAGGCGGATACGAACAT-3′	556	[5]
	5′-CACAGGGCAATTGCTTACCT-3′		
Aerobactin	5′-CTGTCGGCATCGGTTTTATT-3′	531	[5]
	5′-TGGCGTGTCGATTATTACCA-3′		
*Alls*	5′-CCGAAACATTACGCACCTTT-3′		[5]
	5′-ATCACGAAGAGCCAGGTCAC-3′		
*Kfu*	5′- ATAGTAGGCGAGCACCGAGA-3′		[5]
	5′-AGAACCTTCCTCGCTGAACA-3′		

### Multilocus sequence typing (MLST)

MLST were performed according to Turton et al., [4]. Sequences of seven housekeeping genes were obtained for isolates from liver abscess patients and carriers. Sequence information was compared with that available from the MLST website (http://www.pasteur.fr/mlst/) developed by Keith Jolley. Alleles and sequence types (STs) were assigned accordingly. Sequences of any alleles that were not in the database were submitted to the curator and a new allele number obtained. A difference in two or more alleles was considered to indicate that the sequence types being compared were unrelated.

#### Fluorescence labeling of bacteria

Labeling was performed as previously described [18]. The KP isolate and control suspensions were individually incubated overnight at 37°C. The concentration was approximated using photospectrometry (Olympus, US). The percentage of bacterial viability in an aliquot of each population was determined by quantitative plate counting. The FITC-labeled bacteria were resuspended at a concentration of 2 × 10^8^ cells/mL in PBS, divided into equal volumes, and stored at −70°C. Aliquots were thawed just prior to use.

#### Phagocytosis assay

Phagocytosis was measured using a standard assay. Normal human serum pooled from healthy volunteers was divided into equal volumes and stored at −70°C. Serum was thawed immediately prior to use and stored on ice until added to the phagocytosis assay. Briefly, for the assay, 100 μL of a neutrophil suspension (representing 1 × 10^6^ cells), 100 μL of freshly thawed pooled normal human serum (10% v/v opsonization), and 600 μL PBS was added to sealable 10 × 75 mm Falcon™ polypropylene tubes (BD, Franklin Lakes, NJ). The suspension was pre-warmed with shaking for 5 min at 37°C. Multiple volumes of 200 μL FITC-labeled bacteria (representing 4 × 10^7^ colony forming units [cfu]/mL) were added to 800 μL to produce a final volume of 1.0 mL. Each tube was capped and incubated in a shaking water bath at 37°C with continuous agitation for 15 min. An unincubated tube served as the 0-min time point. At each designated time, samples were removed and placed in an ice bath. The cells in each suspension were removed by centrifugation at 250 g for 6 min, and the cell pellet was resuspended in 1.0 mL of ice-cold PBS and maintained at 4°C. A 600 μL volume of the suspension was transferred into a new tube, and ethidium bromide was added to a final concentration of 50 mg/L before measurement. Excess ethidium bromide was used to suppress the extracellular fluorescence. Bacteria that were not localized in neutrophils appeared red in color upon microscopic examination (see below).

#### Phagocytosis assay using flow cytometry

A FACScan, emitting an argon laser beam at 488 nm (Becton Dickinson Immunocytometry Systems, San Jose, Calif.), was used to detect FITC fluorescence. The sideway scatter (SSC) threshold was 52. The detector was set at E00, 350, and 427 for forward scatter (FSC), SSC, and fluorescence 1 (FL1-H, green), respectively. Fluorescence values were collected after gating the detector on the FSC and SSC combination. A total of 10,000 cells were processed using the Cellquest version 1.0 software. Fluorescence distribution data collected using a logarithmic amplifier was displayed as single histograms for FL1-H. By processing unstained and FITC-stained bacterial phagocytosis mixtures, the boundary of positive and negative fluorescence was determined. The percentage of ingested bacteria was assessed after the addition of ethidium bromide.

#### Susceptibility to serum killing

Serum bactericidal activity was measured using the method of Hughes et al. [19] as modified by Podschun et al. [20]. The viable bacterial concentration was adjusted to 1 × 10^6^ colony forming units/mL. Twenty-five microliters of bacteria were added to 75 μL of pooled human sera contained in a 10 × 75 mm Falcon polypropylene tube (BD Biosciences, Franklin Lakes, New Jersey). Tubes were agitated for 0, 60, 120, or 180 min. To determine the number of viable bacteria after exposure to serum, an aliquot of each bacterial suspension was removed at the designated time point, diluted 10-fold by addition of Mueller-Hinton broth, plated on Mueller-Hinton agar, and assayed as described immediately below.

Results were expressed as percentage of inoculums, and responses in terms of viable counts were graded from 1–6 as described previously [20]. Each strain was tested at least three times. A strain was considered serum resistant or serum sensitive if the grading was the same in all experiments. Each isolate was classified as highly sensitive (grades 1 or 2), intermediately sensitive (grades 3 or 4), or resistant (grades 5 or 6).

### Mice lethality test

In determination of LD50 in mice*,* six mice were used as a sample population for each bacterial concentration. Bacterial concentration was calculated by cell forming unit (cfu). Intraperitoneal (i.p.) injection was used to assess virulence. Mice used in this study was approved by animal used committee with NHRI-IACUC-103014-A. Symptoms and signs of infection were observed for 14 days. Survival of the inoculated mice was recorded and the LD50 was calculated using SigmaPlot version 7.0 from SPSS Inc. (Chicago, IL).

## Results

### MLST profiles of isolates from Hong Kong, Singapore and Taiwan

**Figure 1:**
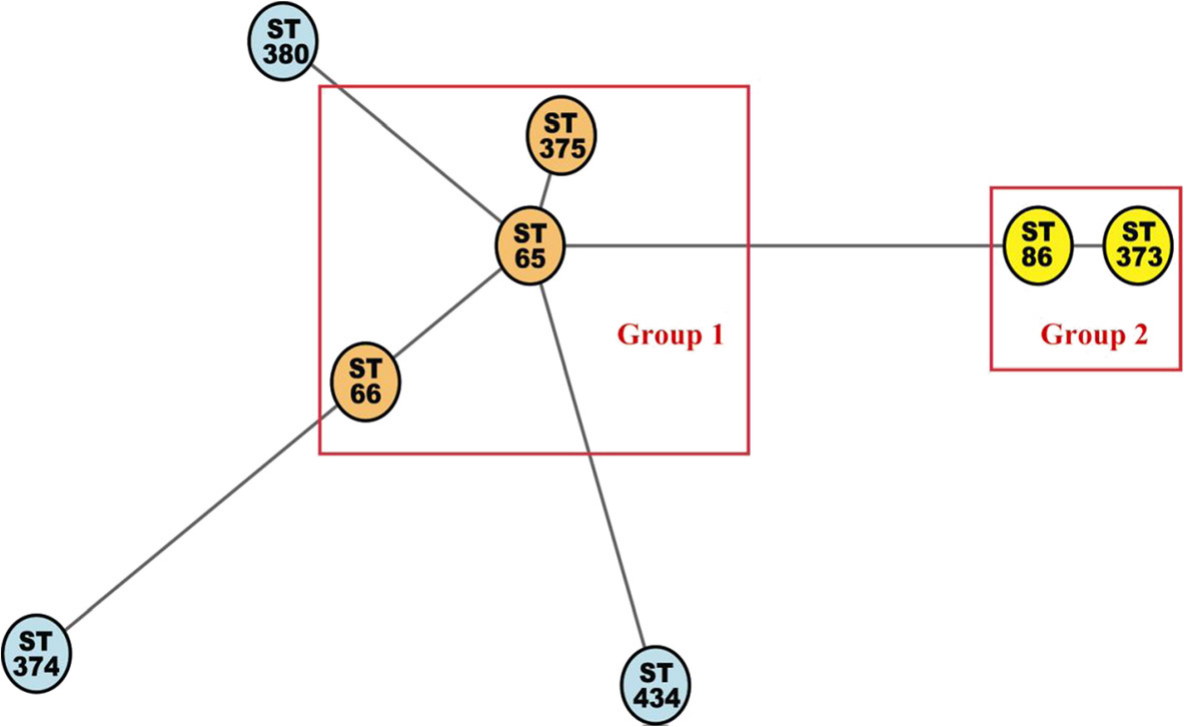
**Minimum-spanning tree of 26 isolates of KP serotype K2 strains generated by MLST allelic data (analysis in MLST website).** ST number, Orange indicates ST group 1; yellow, ST group 2; blue, singleton.

A total of 26 serotype K2 isolates were confirmed by serotyping and PCR and selected for this study. Fifteen and 11 KPs were isolated from liver abscess patients and stool of non-infectious carriers (Table 2) respectively. Eight different MLSTs were identified including ST 65, 66, 86, 373, 374, 375, 380, and 434. Two major MLST groups, ST-65-like and ST86-like groups were obtained based on minimum-spanning tree analysis (Figure 1). ST373 (a single locus variant, SLV to ST86) and ST374 were new ST types found in this study (Table 2). The ST types of liver abscess isolates obtained from Hong Kong were more diverse. There was no specific distribution of ST types between those isolated from liver abscess and from stool carriage.

**Table 2:** MLST of serotype K2 isolates from liver abscess patients and from stool carriage of non-liver abscess subjects in Hong Kong, Singapore and Taiwan

Group*	**ST type (no. of isolates)**	**No. of isolates**						**Allelic profiles**						
		**HK**^**†**^		**SG**		**TW**		***gapA***	***inf***	***mdh***	***pgi***	***pho***	***rpoB***	***tonB***
		**LA**^¶^	**ST**	**LA**	**ST**	**LA**	**ST**							
1	65 (8)	2	2	1		2	1	2	1	2	1	10	4	13
1	375 (1)						1	43	1	2	1	10	4	13
1	66 (2)			2				2	3	2	1	10	1	13
2	86 (11)	1	2	2	1	2	3	9	4	2	1	1	1	27
2	**373 (1)**					1		9	4	2	26	1	1	27
3	**374 (1)**		1					2	3	58*	37	10	27	9
4	380 (1)	1						2	1	1	1	1	4	19
5	434 (1)	1						2	3	2	4	9	4	118

**Figure 2:**
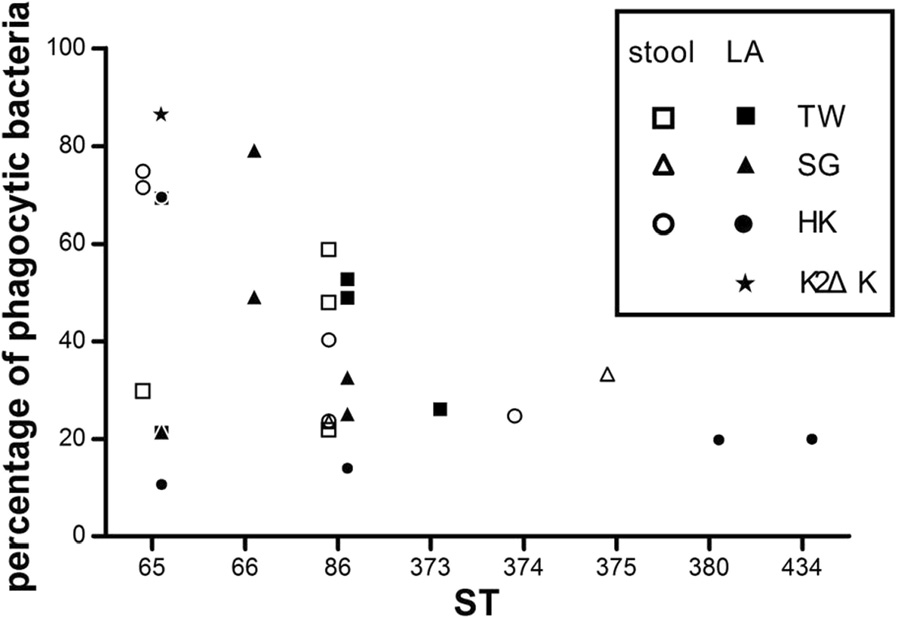
**Neutrophil phagocytosis of all K2 *K. pneumoniae *** **isolates.**

*Grouping was referred to the results obtained from minimum-spanning tree (Figure 2). 373 and 375 were single locus variant to ST86 and 65.

^†^Isolates from: HK, Hong Kong; SG, Singapore; TW, Taiwan.

^¶^Isolates from: LA, liver abscess; ST, stool carriage.

NA: Not available; Bold: New ST types in this study.

### Serum killing resistance and neutrophil phagocytosis of all K2 *K. pneumoniae* isolates

Serum killing resistant and susceptible isolates were found in both ST65-like and ST86-like isolates. Although liver abscess isolates were generally more resistant (11/15 isolates) to serum killing, there was no specific distribution of serum killing resistant or susceptible ST types between stool carriage and liver abscess isolates (Table 3).Liver abscess and carriage isolates had variable susceptibility to phagocytosis. Likewise, there was no difference between the two major MLST types, ST65-like and ST86-like (Figure 2). Although ST other than ST65-like and ST86-like were all more resistant to phagocytosis, there was only one isolate for each type.

**Table 3:** Serotype K2 isolates from stool carriage and liver with different MLSTs in against serum complement killing

**Source**	**ST type (No. of isolates, N)**	**Serum complement killing**					
		**Non-susceptible**^**#**^			**Susceptible**		
		HK*	SG*	TW*	**HK**	**SG**	**TW**
Stool carriage	65 (3)			1	2		
	86 (6)	2	1	2			1
	374 (1)				1		
	375 (1)			1			
Liver abscess	65 (5)	2		1		1	1
	66 (2)		2				
	86 (5)	1	1	2		1	
	373 (1)			1			
	380 (1)	1					
	434 (1)				1		

*HK, Hong Kong; SG, Singapore; TW, Taiwan. ^#^Intermediately sensitive or resistant to serum killing.

### Comparative analysis with respect to mouse lethality, susceptibility to neutrophil phagocytosis, serum killing and virulence-associated genes

PCR for virulence associated genes revealed that all isolates contained *rmpA* while none harbored *allS* gene. The prevalence of the aerobactin gene and *kfu* in all K2 isolates were 25/26 (96%) and 3/26 (11.5%) respectively. Fifteen isolates from either liver or stool carriage showed resistance to both neutrophil phagocytosis and serum killing. Twelve were hypervirulent with LD_50_ ≤ 10^2^ CFU. Two isolates with LD_50_ equal to 1.0 × 10^3^ CFU and 1 with LD_50_ 5.5 × 10^3^ CFU were also defined as virulent strains. Four isolates showed susceptibility to both neutrophil phagocytosis and serum killing. These four isolates demonstrated lethality with LD_50_ that ranged from <3.7 × 10^2^ - 2.2 × 10^6^ CFU in the infection model. These two parameters alone were not able to reflect the virulence of the strains. Other factors that may contribute to virulence are not further assessed in this study. One ST86 strain was hypervirulent with a mouse lethality LD_50_ of <3.7 × 10^2^ CFU, suggesting other unknown and major virulent factor might contribute to the hypervirulence. Hypervirulent strains could be found in both liver and stool carriage isolates. Among the virulent associated factors tested in this study, no other combination of parameters could be used to predict virulence in the mice lethality model. The presence of virulence associated genes did not correlate with increasing mice lethality.

## Discussion

Serotype K2 *K. pneumoniae* is the second most prevalent serotype next to serotype K1 as a cause of pyogenic liver abscess and is also frequently reported in community acquired pneumonia [21]. Although previous investigations have observed that serotype K2 isolates are not a significant risk factor for septic ocular or CNS complications [11], and a “Medline” search revealed few reports in patients of non-Asian descent with KP serotype K2 liver abscesses [21,22], the virulence of serotype K2 should not be underestimated [23]. In a study of KP liver abscess in China, a comparable frequency of isolation between serotype K1 (43%) and K2 (37%) was observed [15] indicating the possible difference in geographic distribution. KP invasive syndrome due to serotype K2 has also been reported in Asian patients [22]. In a French study of severe and fatal infections due to KP, isolates from the fatal cases were all of capsular serotype K2 [21]. Of major concern was a recent report on an increasing antimicrobial resistance among these hypervirulent strains of KP [24]. The clinical impact of these hypervirulent strains with multiple drug resistance should be further investigated.

Epidemiology studies showed that a major MLST type, ST-23, was associated with serotype K1 liver abscess [2,4,9,25]. Few MLST-based studies have specifically focused on serotype K2 isolates except for a recent study in Taiwan where MLST was performed in KP strains causing different infections [25], Laio et al., [25] have found more diversity of STs in serotype K2 compared with K1. Eleven STs were observed in their collection. Unlike serotype K1, it seems that no major ST type could be identified as a cause of liver abscess. However, few isolates including ST65 (*n* = 3) and one each of ST373, and ST375 were identified in the study of Liao et al., [25]. In the present study, 8 different MLSTs were identified among serotype K2 isolates from liver abscesses and stool carriage. We observed that two major MLSTs’ groups, ST6- and ST86-like groups, were responsible for most of the liver abscess cases (11/15). These two major MLST groups were also the most prevalent MLSTs in stool carriage isolates. Previous study has shown that carriage of isolates in the stool is one of the predisposing factors for liver abscess [26]. A high prevalence of these two major MLST groups in both liver abscess and stool carriage isolates supports this and suggests that the colonization of virulent type KP is an important step for the development of liver abscess. Although ST86 was the most frequently isolated ST type next to ST65, none were isolated in the study by Liao et al., [25].

In the virulence analysis, anti-phagocytosis and -serum killing could be used to predict hyper-virulence (LD_50_ < 10^3^ CFU) or relative non-virulence (LD_50_ ≥ 10^5^ CFU) in serotype K2 isolates (Table 4). A ST86 stool carriage isolate with susceptibility to both neutrophil and serum killing had an LD_50_ equal to 10^2^. This isolate carried aerobactin but lacked *kfu* and *allS*. An ST-65 isolate with an identical virulence profile had a low virulence by mice lethality (LD_50_ at 2.2 × 10^6^), indicating other unknown factor(s) may also play a role in the virulence (Table 4). Virulence associated *kfu* was only identified in the non major types of ST 373, 375 and 380. This chromosomal gene is involved in iron uptake and has been described in most tissue invasive *K. pneumoniae*[12]. Our data shown that the two major ST types, including both hypervirulent and non-hypervirulent isolates, did not carry this gene. The presence of this chromosomal gene in only minor ST types may reflect the difference in genetic background among serotype K2 strains. Whether the presence of this gene determines the fitness for causing disease needs further investigation. The absence of *allS* in all serotype K2 *K. pneumoniae* match the observation that this is only found in serotype K1 strains [5].

**Table 4:** Virulence analysis for all 26 isolates by combining results obtained from presence of virulent associated genes, phagocytosis, serum complement killing and mice lethality

**Strain no.**	Source*	**MLST**	**Phagocytosis**	**Serum**	***kfu***	**Aerobactin**	**LD**_**50 **_**(cfu)**	**Virulence**^**†**^
1	Carriage	ST65	S	S	-	-	1.2 × 10^5^	+
2	Liver	ST65	S	S	-	+	2.2 × 10^6^	-
3	Carriage	ST65	S	R	-	+	<1.9 × 10^2^	+++
4	Liver	ST65	S	R	-	+	<1.3 × 10^2^	+++
5	Liver	ST65	R	S	-	+	<1.1 × 10^2^	+++
6	Liver	ST65	R	R	-	+	<2.1 × 10^2^	+++
7	Liver	ST65	R	R	-	+	1.0 × 10^2^	+++
8	Carriage	ST65	R	R	-	+	<2 × 10^2^	+++
9	Liver	ST66	R	R	-	+	1.8 × 10^2^	+++
10	Liver	ST66	R	R	-	+	1.0 × 10^3^	+
11	Carriage	ST86	S	S	-	+	<3.7 × 10^2^	+++
12	Liver	ST86	R	S	-	+	3.4 × 10^4^	+
13	Liver	ST86	S	R	-	+	<1.0 × 10^2^	+++
14	Carriage	ST86	S	R	-	+	5.3 × 10^4^	+
15	Liver	ST86	R	R	-	+	5.5 × 10^3^	+
16	Liver	ST86	R	R	-	+	1.0 × 10^2^	+++
17	Liver	ST86	R	R	-	+	<1.0 × 10^2^	+++
18	Carriage	ST86	R	R	-	+	<1.0 × 10^2^	+++
19	Carriage	ST86	R	R	-	+	<1.0 × 10^2^	+++
20	Carriage	ST86	R	R	-	+	<1.0 × 10^2^	+++
21	Carriage	ST86	R	R	-	+	1.0 × 10^3^	
22	Liver	ST373	R	R	-	+	<3.4 × 10^2^	+++
23	Carriage	ST374	R	S	+	+	1.5 × 10^3^	+
24	Carriage	ST375	R	R	+	+	2.8 × 10^2^	+++
25	Liver	ST380	R	R	+	+	<1.9 × 10^2^	+++
26	Liver	ST434	S	S	-	+	2.6 × 10^4^	+

*All isolates were carrying *rmpA*. No isolate was carrying *all*S. ^†^Interpretation of virulence was referred to reference [2]. (+++) = hypervirulent strains with an LD50 of less than 1 × 10^3^ colony-forming units (CFU) are more likely to induce complications in mice. (+) = virulent strains with a 50% lethal dose (LD50) between ≥1 × 10^3^ and ≤1 × 10^5^ CFU are less likely to induce complications in mice. (−) = non-virulent strains with an LD50 of 1 × 10^6^ CFU of greater (do not cause complications).

In summary, anti-phagocytosis and resistance to serum killing are the two main parameters that predict hypervirulence in serotype K2 isolates. Other yet unknown factor(s) may contribute to virulence, as has been observed in this study. Unlike serotype K1 KP-LA which are usually ST-23, ST65-like and 86-like are the two major MLST types among serotype K2 isolates from Singapore, Hong Kong and Taiwan.

## Competing interests

The authors declared that they have no competing interests.

## Authors’ contributions

JCL, LKS MI,TSK designed study and drafted the manuscript, CPF, JCL, NL, FYC, MI, TSK enrolled the patients and collected the isolates in this study. MI, TSK and LKS proof read and edited the manuscript. All authors read and approved the final manuscript.

## Funding

This study was supported by research grants from National Science Council (NSC102-2314-B016-013-MY-3) and National Health Research Institutes.
